# Time to death and risk factors associated with mortality among COVID-19 cases in countries within the WHO African region in the early stages of the COVID-19 pandemic

**DOI:** 10.1017/S095026882100251X

**Published:** 2022-02-18

**Authors:** Benido Impouma, Alice L. J. Carr, Alexander Spina, Franck Mboussou, Opeayo Ogundiran, Fleury Moussana, George Sie Williams, Caitlin M. Wolfe, Bridget Farham, Antoine Flahault, Claudia Codeco Tores, Jessica L. Abbate, Flávio Codeço Coelho, Olivia Keiser

**Affiliations:** 1 World Health Organization, Regional Office for Africa, Brazzaville, Congo; 2 Institute of Global Health, University of Geneva, Geneva, Switzerland; 3 Institute of Biomedical and Clinical Science, University of Exeter Medical School, Exeter, UK; 4 University of Exeter Medical School, Heavitree Road, Exeter, UK; 5 College of Public Health, University of South Florida, Tampa, Florida, USA; 6 Fundação Oswaldo Cruz, Rio de Janeiro, Brazil; 7 UMI TransVIHMI (Institut de Recherche pour le Développement, Institut National de la Santé et de la Recherche Médicale, Université de Montpellier), Montpellier, France; 8 The GRAPH Network, Geneva, Switzerland; 9 Fundação Getulio Vargas, Rio de Janeiro, Brazil

**Keywords:** Comorbidities, COVID-19, mortality, risk factors, SARS-CoV-2

## Abstract

This study describes risk factors associated with mortality among COVID-19 cases reported in the WHO African region between 21 March and 31 October 2020. Average hazard ratios of death were calculated using weighted Cox regression as well as median time to death for key risk factors. We included 46 870 confirmed cases reported by eight Member States in the region. The overall incidence was 20.06 per 100 000, with a total of 803 deaths and a total observation time of 3 959 874 person-days. Male sex (aHR 1.54 (95% CI 1.31–1.81); *P* < 0.001), older age (aHR 1.08 (95% CI 1.07–1.08); *P* < 0.001), persons who lived in a capital city (aHR 1.42 (95% CI 1.22–1.65); *P* < 0.001) and those with one or more comorbidity (aHR 36.37 (95% CI 20.26–65.27); *P* < 0.001) had a higher hazard of death. Being a healthcare worker reduced the average hazard of death by 40% (aHR 0.59 (95% CI 0.37–0.93); *P* = 0.024). Time to death was significantly less for persons ≥60 years (*P* = 0.038) and persons residing in capital cities (*P* < 0.001). The African region has COVID-19-related mortality similar to that of other regions, and is likely underestimated. Similar risk factors contribute to COVID-19-associated mortality as identified in other regions.

## Introduction

In late December 2019, a novel coronavirus identified as severe acute respiratory syndrome coronavirus-2 (SARS-CoV-2) was detected in several cases of pneumonia in Wuhan City, Hubei Province, China [1]. Within a month several countries were reporting cases of the disease, named by the World Health Organisation (WHO) as coronavirus disease 2019 (COVID-19), with the deployment of testing resources demonstrating the rapid spread across international borders.

Although mortality from SARS-CoV-2 is lower, transmission is higher when compared to other emerging coronaviruses causing severe acute respiratory syndrome (SARS) epidemics over the last two decades [2–4]. As of February 2021, the global case fatality ratio (CFR) for SARS-CoV-2 was estimated at 2.3% [5], compared to 9.7% for SARS-CoV which emerged in late 2002 [3], and 34% for Middle East respiratory syndrome coronavirus (MERS-CoV) which emerged in 2012 [4]. The basic reproductive rate (*R*_0_) for SARS-CoV-2 is 2.5 compared to 2.4 for SARS-CoV and 0.69 for MERS-CoV [2, 6]. Although of similar *R*_0_ to SARS-CoV based on available data, SARS-CoV-2 has spread rapidly to all continents.

Initial cases of COVID-19 were detected in Africa in February 2020, introduced by travellers from Europe into Egypt and Algeria [7]. The outbreak in the WHO African region evolved rapidly, and by 13 May 2020, all 47 countries had been affected [8]. Confirmed case numbers began to increase from April 2020, reaching a peak by the end of July 2020, which then declined through August and September 2020, before increasing again during November and December 2020 [5].

As of 24 February 2021, 12 months after the notification of the first laboratory-confirmed COVID-19 case, a cumulative total of 2 811 106 confirmed cases and 71 159 deaths have been reported in the African region, with the CFR estimated at 2.5% [5]. This represents 2.5% of cases globally and 2.8% of deaths [5]. This proportion of cases is low when compared to the Americas (45%) and Europe (34%) [5]. The reason for this disparity may be due to low testing performance within the African region. As of 24 February 2021, 25904273 SARS-CoV-2 tests (molecular and antigen) across 43 African region countries were performed, representing 241.5 tests per 10 000 population. Only 41.8% (18 out of 43) of the countries assessed surpassed the effective testing rate (10 tests per 10 000 population per week) between 28 January and 24 February 2021 (data unpublished). Other factors contributing to this disparity have been suggested including early implementation of travel restrictions, border closures, lockdown measures including curfews and school closures, a younger population, genetics, lower comorbidities rates, possible trained immunity or immunomodulation, suboptimal testing, and favourable climate [9–11].

While several studies documented the occurrence of deaths in COVID-19-confirmed patients, there are limited studies in the WHO African region addressing mortality burden and the risk factors associated with COVID-19 [10, 12]. The purpose of this study is to describe the risk factors associated with mortality among COVID-19 cases reported in the WHO African region in the early stages of the COVID-19 pandemic between 21 March and 31 October 2020, to understand if these differ from other regions, and to inform future measures that should be taken by public health authorities to address and mitigate the impact in the WHO African region.

## Methods

### Study design

We conducted a retrospective cohort study of deaths associated with confirmed COVID-19 cases reported by Member States in the WHO African region between 21 March and 31 October 2020. The time period was chosen to maximise the number of countries with complete reports.

### Case definitions

A confirmed case of COVID-19 was defined as a person with a positive Nucleic Acid Amplification Test (NAAT) or a person with a positive SARS-CoV-2 Ag-RDT *and* meeting either the probable case definition or suspected criteria as per the WHO guideline, or an asymptomatic person with a positive SARS-CoV-2 Ag-RDT *and* who is a contact of a probable or confirmed case [13]. A COVID-19 death is defined as a death resulting from a clinically compatible illness in a probable or confirmed COVID-19 case, unless there is a clear alternative cause of death that cannot be related to COVID-19 disease (e.g. trauma) [13].

### Data source

The primary data source was the regional linelist of confirmed COVID-19 cases, a database containing key information about each confirmed case reported to the WHO Regional Office for Africa (WHO AFRO) by its Member States per the reporting requirements of the International Health Regulations (2005) [14]. Variables captured include unique identification, date of reporting, age, sex, location (administrative levels 1 and 2), case classification, occupation, health worker status, date of symptom onset, presence of symptoms, laboratory test result, date of sample collection, date of laboratory result, date of death, date of discharge, patient outcome, current inpatient status, and presence and description of comorbidity.

### Exclusion criteria

All countries' cases in the WHO AFRO regional linelist were eligible for inclusion. We excluded cases reported before 21 March or after 31 October 2020 as well as cases missing information on patient outcome, key dates (e.g. outcome date if died or laboratory result date), age or sex. Cases with laboratory result dates after outcome dates were also excluded.

### Data cleaning

We identified confirmed cases using the case classification variable. Where this was unavailable, we used the laboratory test result. Start dates were defined for individuals as the date of confirmed laboratory result; where this was unavailable the sample collection date was used. This was deemed to be more complete and reliable than either symptom onset or sample collection date in isolation. End dates for individuals were defined as the earliest occurring date where the individual was reported dead or recovered; in the absence of recovery date, the maximum date of observation was used (31 October 2020). Observation time was calculated as the difference between start and end dates in days. Patients were identified as recovered if the patient outcome variable contained prespecified words related to recovery. Where patient outcome was unavailable, we used current inpatient status. Patient outcome was dichotomised to ‘alive’ or ‘dead’.

We coded the following exposure variables: healthcare worker status, residence in capital city status and comorbidity status and types. Healthcare worker status was identified if the free-text occupation variable contained prespecified key words related to healthcare. Healthcare worker status was defined as ‘Not Reported’ in the absence of the occupation variable. Capital city residence was identified if the free-text location variable (administrative level 2) contained the country's capital city. Capital city residence was defined as ‘Not Reported’ in the absence of the free-text location variable. Comorbidity types were identified if the comorbidity free-text variables contained prespecified key words for each comorbidity type of interest (diabetes, asthma, hypertension, cancer, renal disease, cardiovascular disease, obesity, tuberculosis, sickle cell disease, chronic pulmonary or other). Comorbidity type was defined as ‘Not Specified’ if presence was indicated with ‘Yes’ but no further description was provided. Comorbidity presence was categorised as ‘Yes’ if a defined comorbidity type was detected in the previous step, ‘No’ if indicated so in free-text description or if the free-text description contained non-comorbidities and ‘Not reported’ in the absence of the comorbidity free-text variables. These exposure variables were dichotomised as ‘Yes’ and ‘No/Not Reported’.

Based on age at reporting, we created a dichotomous age variable (⩾60 years) and an age group variable in years as follows: ≤10, 11–20, 21–30, 31–40, 41–50, 51–60, 61–70 and 70+.

### Data analysis

#### Incidence and case fatality ratios

We calculated the incidence per 100 000 population as the number of confirmed cases divided by the population multiplied by 100 000. We sourced population data for each country in 2020 from the United Nations World Population Prospects [15] and summed these to calculate the overall.

We calculated the CFR, the proportion of confirmed cases that died due to the consequences of COVID-19 [16], by dividing the cumulative number of deaths by the cumulative number of confirmed cases. We stratified results by country, age group, sex, comorbidity (and number of comorbidities), residence and healthcare worker status to ascertain the most affected categories.

#### Age and sex distributions

To determine the distribution of cases and deaths stratified by age group and sex, we calculated the proportion in each group of the total number, and plotted this in an age-sex pyramid. We additionally plotted the CFR by age group and sex.

#### Comorbidities

In addition to describing CFR by individual comorbidities, we conducted a combination analysis for the comorbidities/condition of interest (as defined in data cleaning). This investigated the most frequently occurring combinations of comorbidities among all confirmed cases and among those who died. We also investigated the exposure–response relationship between increasing number of comorbidities and mortality compared to those without comorbidity using weighted Cox regression (see regression details in risk factors for death section).

#### Risk factors for death

We used Cox regression to compute hazard ratios and corresponding 95% confidence intervals to investigate associations between mortality and several dichotomous exposure variables (healthcare worker status, residence in capital city status, comorbidity status) and age as a continuous variable. These variables were chosen based on associations with increased mortality identified in previous literature.

Given the low number of variables included in the univariate analysis, we decided *a priori* to include all variables in the multivariable analysis regardless of significance level in univariate analyses. We excluded pregnancy status from multivariable analysis, given that this is only relevant to females, leading to data separation.

We investigated all variables for confounding and effect modification using Mantel–Haenszel statistics and associated Woolf's tests, in order to identify necessary interaction terms.

The model proportional hazards assumption was tested using scaled Schoenfeld residuals, with non-linearity assumptions assessed visually. As the hazards were found to be non-proportional, we present the average hazard ratio and corresponding 95% CI calculated using weighted Cox regression [17].

#### Time to death

At a population level, the case fatality was not above 50%, thus it was not possible to calculate median survival times using Cox regression. Instead, we calculated the medians and interquartile ranges of observation time in days among those who died, stratified by exposure variables and compared the distribution of these times using Kruskal–Wallis tests.

All analyses were two-tailed, with a significance level of 0.05, and carried out using R statistical software version 3.6.1 (Foundation for Statistical Computing, Vienna, Austria).

## Results

### Inclusion

**Fig. 1.**
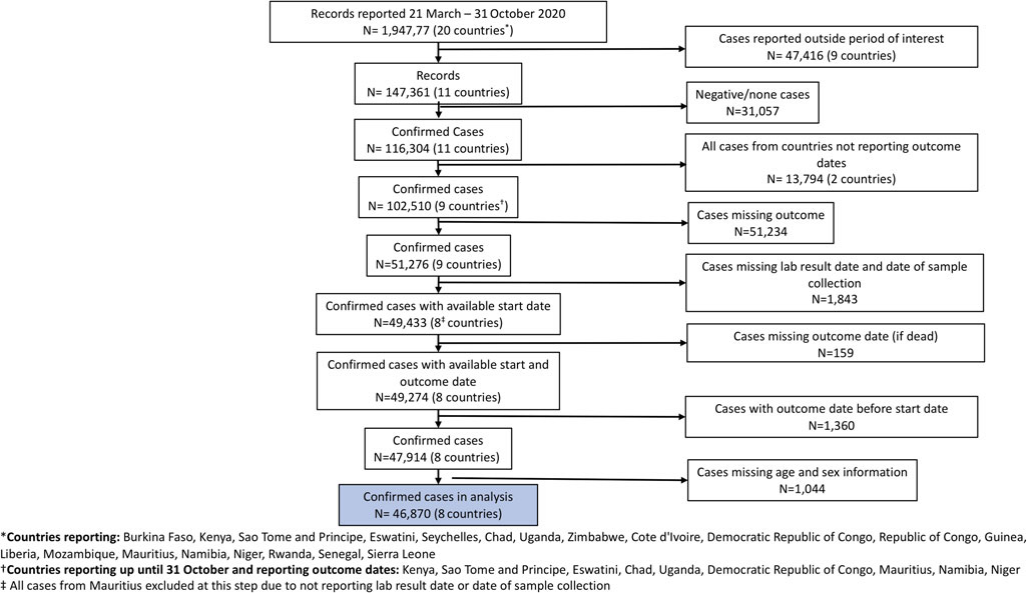
Flowchart of inclusion pathway for cases.

Of the 194 777 COVID-19 cases reported in 20 countries of the WHO African region from 21 March to 31 October 2020, we selected 46 870 cases (24%) for the study with a total observation time of 3 959 874 person-days. The cases meeting our selection criteria were reported from eight WHO Member States in the WHO African region. Cases from WHO Member States who stopped reporting before 31 October 2020 (*n* = 47 416 from nine countries) and who did not report outcome dates (*n* = 31 678 from two countries) were excluded. Reported cases with negative results (*n* = 13 173), missing outcomes and incomplete dates (*n* = 54 596) and missing age and sex information (*n* = 1044) were also excluded (Fig. 1). Characteristics and missingness for variables of interest among confirmed cases are outlined in Supplementary Table S1.

A comparative summary for 116 304 COVID-19 cases reported between 21 March and 31 October 2020, for 11 WHO Member States reporting up until and including 31 October, prior to exclusion due to covariate data availability, is outlined in Supplementary Table S2.

### Incidence and case fatality ratio

The overall incidence among the eight WHO Member States included in this analysis was 20.06 per 100 000. Namibia had the highest incidence (49.43 per 100 000), followed by Sao Tome and Principe (42.02 per 100 000) and Eswatini (11.21 per 100 000). Uganda (*n* = 12 126) and the Democratic Republic of Congo (*n* = 10 274) reported high numbers of cases, but had a relatively low incidence (9.25 and 1.15 per 100 000, respectively) (Table 1).

**Table 1.** Summary measures for COVID-19 cases reported between 21 March and 31 October 2020 by Member States of the WHO African region included in analysis (*N* = 46 870)

Country	Cases^a^ (*N*)	Recovered^b^ (*n*)	Dead (*n*)	Alive^c^ (*n*)	CFR (%)	Healthcare worker cases (*N*)	Healthcare worker deaths (*n*)	Healthcare worker cases (%)	CFR among healthcare workers (%)	Incidence per 100 000
Democratic Republic of Congo	10 274	532	100	10 174	0.97	208	4	2.02	1.92	1.15
Kenya	7421	6865	554	6867	7.47	269	11	3.62	4.09	1.38
Namibia	12 560	605	34	12 526	0.27	532	2	4.24	0.38	49.43
Niger	1169	1026	51	1118	4.36	178	0	15.23	0	0.48
Sao Tome and Principe	921	0	4	917	0.43	0	0	0		42.02
Eswatini	1301	1289	12	1289	0.92	117	2	8.99	1.71	11.21
Chad	1098	16	8	1090	0.73	1	0	0.09	0	0.67
Uganda	12 126	0	40	12 086	0.33	76	0	0.63	0	2.65
Total	46 870	10 333	803	46 067	1.71	1381	19	2.94	1.38	20.06

a Confirmed cases meeting selection criteria reported 21st March–31st October 2020.

b Recovery status missing for Sao Tome and Principe and Uganda.

c Recovered cases are included in alive count. Alive and dead counts sum to total cases.

A total of 803 deaths of confirmed COVID-19 cases included in our analysis were reported from the eight WHO Member States. Kenya reported the largest proportion of these (70%, *n* = 554) and also had the highest CFR (7.47%). Niger was the only other member state to have CFR >1% (4.36%), with all others having CFR <1% (Table 1). The overall CFR among the eight WHO Member States was 1.71%. Among the 116 304 COVID-19 cases from 11 WHO Member States, prior to exclusion due to covariate data availability, the incidence was 43.25 per 100 000. The number of deaths totalled 2166 with the overall CFR at 1.86% (Supplementary Table S2).

Healthcare workers made up 2.9% (*n* = 1381) of included cases and had a lower CFR at 1.38%. Namibia reported the highest absolute number among healthcare workers (*n* = 532), but Niger had the highest proportion at 15.2% (*n* = 178). CFR among healthcare workers varied by country (min: 0%, max: 4.09%) with the highest CFR reported by Kenya (Table 1).

### Age and sex distribution

**Fig. 2.**
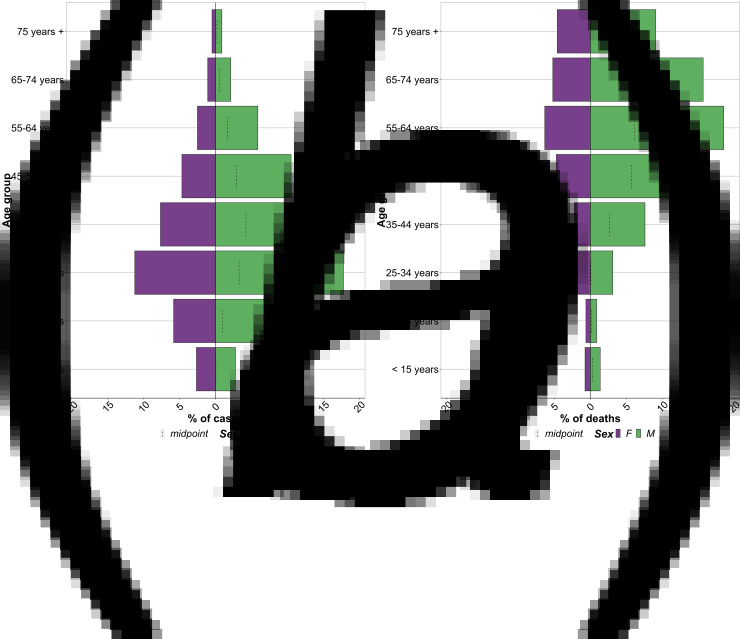
(a) Age-sex pyramid of confirmed cases (a) and of deaths among confirmed cases (b) reported in eight Member States of the WHO African region included in analysis between 21 March and 31 October 2020 (*N* = 46 870, *N* = 803 respectively).

**Fig. 3.**
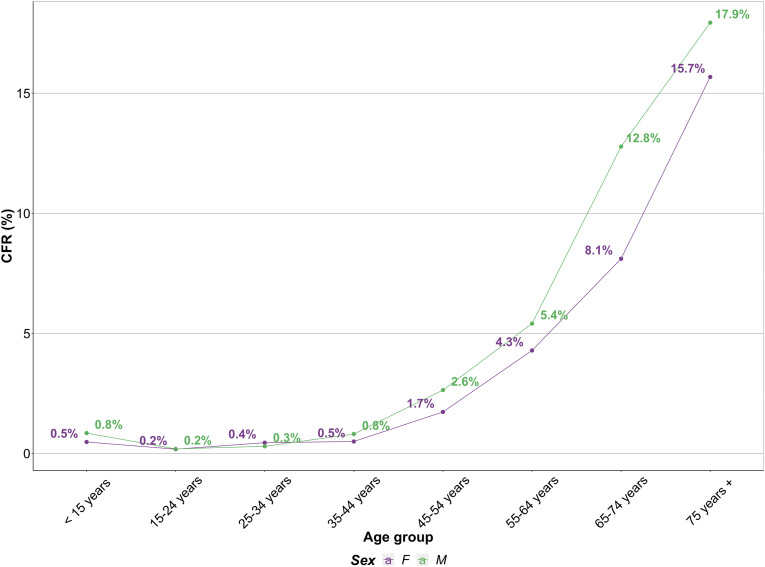
Age and sex-specific case fatality ratio among confirmed cases reported in eight Member States of the WHO African region included in analysis between 21 March and 31 October 2020 (*N* = 46 870).

Among confirmed COVID-19 cases included in our study, 52% occurred in the ages between 21 and 40 years, with the majority of deaths occurring in persons aged over 40 years (84%). There were a higher proportion of male cases compared to females overall (1.7:1) and also within all age groups over 20 years (Fig. 2a). Similarly, deaths were reported twice as often in males, with a notably increased proportion of deaths in all age groups over 30 years of age (Fig. 2b). In the age groups under 50 years, CFR was approximately equal for males and females and remained stable. In the age groups older than 50 years however, the CFR was approximately 0.7 times higher with every 10 years of age in both males and females, and was higher in males in each of the age groups (Fig. 3). For both males and females, the lowest CFR occurred between 11 and 20 years (males: 0.3%, females: 0.2%) with the highest occurring in persons aged 70 years and over (males: 17.7%, females: 13.7%).

Among 1381 healthcare worker cases, 56.6% were female (*N* = 783), with a higher proportion occurring in the age groups below 30 years (33% *vs.* 25% for males). Male healthcare workers had a higher overall CFR (2.17% *vs.* 0.77%) (Supplementary Table S3).

### Comorbidities

**Fig. 4.**
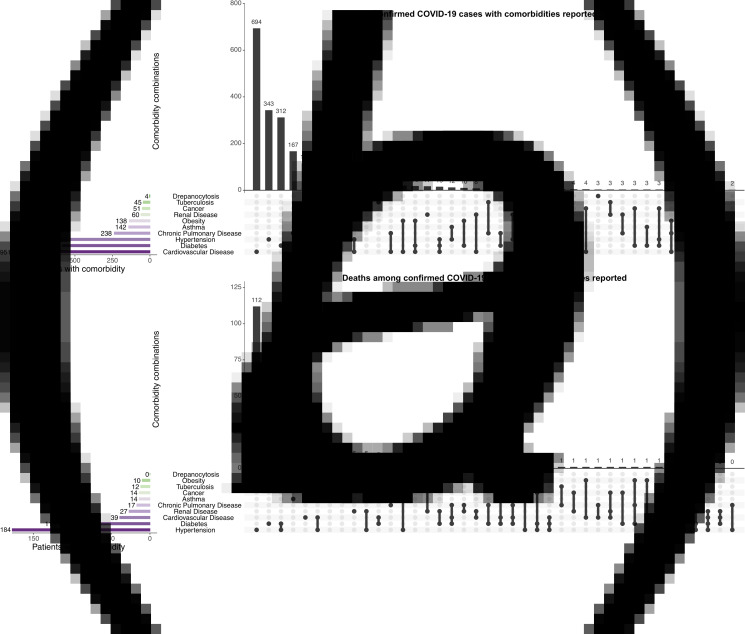
(a) Combination analysis of comorbidities of interest – demonstrating the 40 most common combinations among confirmed cases (a) and confirmed cases that died (b) with comorbidities of interest in eight Member States of the WHO African region included in analysis between 21 March and 31 October 2020 (*N* = 2227, *N* = 310 respectively).

Cardiovascular disease was the most commonly reported comorbidity among confirmed COVID-19 cases included in our analysis, both overall (*n* = 951) and as a single comorbidity (*n* = 694) (Fig. 4a). This means, among those reporting cardiovascular disease, 73% had no other comorbidities. Diabetes (*n* = 585) and hypertension (*n* = 535) were also commonly reported among cases (Fig. 4a); 52% (*n* = 312) and 64% (*n* = 343), respectively, were reported as single comorbidities. While 39 deaths (CFR 4.1%) occurred among those with cardiovascular disease, only nine deaths (CFR 1.3%) occurred in those with isolated cardiovascular disease (Supplementary Table S4).

The highest number of deaths were reported in cases with only hypertension (*n* = 112, CFR 33%) (Fig. 4b). The overall CFR among all cases with hypertension (singularly or in combination) was 34.4% (Supplementary Table S4). Cases with only diabetes reported had the second highest number of deaths (*n* = 50, CFR = 16%) (Fig. 4b) with an overall CFR 20.2% for all cases with diabetes (Supplementary Table S4).

Cases with renal disease had the overall highest CFR (45%), with 27 dead out of a total of 60 (Supplementary Table S4). However, 70% of these deaths occurred in cases with other comorbidities, including renal disease with hypertension (*n* = 6, 20%) and renal disease with diabetes (*n* = 3, 10%) (Fig. 4b). The overall CFR for a further six common comorbidities is outlined in Supplementary Table S4, as well as comorbidities categorised as other, non-specified comorbidities and pregnancy.

The hazard of death significantly increased with increasing number of comorbidities. Cases with one comorbidity from the specified list (excluding those only listed as ‘other’ or non-specified) had 12 times greater hazard of death than those without comorbidities (aHR 11.6 (95% CI 9.87–13.73); *P* < 0.001) (Table 2). The maximum number of comorbidities specified was four, and cases in this group had a 66 times higher hazard of death compared to those without comorbidities (aHR 66.01 (95% CI 28.50–152.42); *P* < 0.001), although this is likely a problem of low numbers, which will give an unrealistically high ratio (Table 2).

**Table 2.** Univariate weighted cox regression for exposure–response relationship comparing increasing number of comorbidities of interest to those without, among confirmed cases with comorbidities of interest in eight Member States of the WHO African region between 21 March and 31 October 2020 (*N* = 46 236)

Number of comorbidities^a^	CFR	*N*	Dead (*N*)	Time (days)	aHR	95% CI	*P*-value
No/not reported	1.05	44 009	463	3 732 259			
1	11.59	1786	207	137 250	11.64	9.87–13.73	<0.001
2	23.16	367	85	28 719	24.14	19.22–30.33	<0.001
3	20.90	67	14	5543	22.30	13.11–37.91	<0.001
4	57.14	7	4	308	66.01	28.59–152.42	<0.001

aHR, average hazard ratio; CI, confidence interval.

a Specific comorbidities identified included diabetes, asthma, hypertension, obesity, cardiovascular disease, tuberculosis, renal disease, drepanocytosis, chronic pulmonary disease and cancer. Other/not specified comorbidity was not included in this analysis.

### Risk factors for death

In univariate regression, males (aHR 1.46 (95% CI 1.27–1.74); *P* < 0.001), increasing age (aHR 1.08 (95% CI 1.07–1.08); *P* < 0.001), persons who lived in a capital city (aHR 1.88 (95% CI 1.63–2.16); *P* < 0.001) and those with one or more comorbidity (aHR 11.89 (95% CI 10.33–13.69); *P* < 0.001) had a higher hazard of death. Healthcare worker status in women (aHR 0.76 (95% CI 0.46–1.21); *P* = 0.248) and pregnancy (aHR 1.06 (95% CI 0.26–4.28); *P* > 0.9) was not significantly associated with hazard of death (Table 3).

**Table 3.** Weighted cox regression for mortality by various characteristics among confirmed cases reported in eight Member States of the WHO African region between 21 March and 31 October 2020 (*N* = 46 870)

					Univariate			Multivariate	
Characteristic	*N*	Dead (*N*^a^)	Time (days^a^)	aHR	95% CI	*P*-value	aaHR	95% CI	*P*-value
Sex (male)	46 870	582	2 565 154	1.49	1.27–1.74	<0.001	1.54	1.31–1.81	<0.001
Age (continuous)	46 870			1.08	1.07–1.08	<0.001	1.08	1.07–1.08	<0.001
Health care worker status	46 870	19	149 076	0.76	0.49–1.21	0.248	0.59	0.37–0.93	0.024
Residence in capital city status	46 870	347	1 662 749	1.88	1.63–2.16	<0.001	1.42	1.22–1.65	<0.001
Comorbidity status	46 870	340	227 615	11.89	10.33–13.69	<0.001	36.37	20.26–65.27	<0.001
Pregnancy	17 012^b^	584	10 910	1.06	0.26–4.28	0.935			
Comorbidity status×age (continuous)							0.97	0.96–0.98	<0.001

aHR, average hazard ratio; CI, confidence interval; aaHR, average adjusted hazard ratio.

a Unweighted counts.

b Females only.

While controlling for other covariates and their interaction in multivariable analysis, males (aHR 1.54 (95% CI 1.31–1.81); *P* < 0.001), increasing age (aHR 1.08 (95% CI 1.07–1.08); *P* < 0.001), persons who lived in a capital city (aHR 1.42 (95% CI 1.22–1.65); *P* < 0.001) and those with one or more comorbidity (aHR 36.37 (95% CI 20.26–65.27); *P* < 0.001) had a higher hazard of death. Being a healthcare worker reduced the average hazard of death by 40% (aHR 0.59 (95% CI 0.37–0.93); *P* = 0.024) (Table 3). For comparison, unweighted hazard ratios for univariate and multivariable Cox regression are outlined in Supplementary Table S5, but should not be interpreted due to violation of the proportional hazards assumption.

### Time to death

The time to death in those that died (*N* = 803) was significantly less for persons aged 60 and over (<60 years, median (IQR): 4 (2, 9) days *vs.* ≥60 years, median (IQR): 5 (2, 10) days; *P* = 0.038) and for those not reporting residing in a capital city (residence in capital, median (IQR): 4 (2, 9) days *vs.* residence outside of capital/not reported, median (IQR): 6 (2, 11) days; *P* < 0.001) (Table 4). Sex, healthcare worker status and comorbidity status did not significantly impact time to death for confirmed COVID-19 cases (Table 4). Median time to death varied by country with a range between 1 and 10.5 days (data not shown).

**Table 4.** Time to death by various characteristics among confirmed cases reported in eight countries between 21 March and 31 October 2020 (*N* = 803)

Characteristic	Dead (*N* = 803)	Time to death (days)^a^	*P*-value^b^
Sex			0.565
Female	221	4 (2, 10)	
Male	582	5 (2, 9)	
Age			0.038
<60	428	5 (2, 10)	
≥60	375	4 (2, 9)	
Health care worker			0.200
No/not reported	784	5 (2, 9.25)	
Yes	19	7 (4, 11.5)	
Residence in capital city			<0.001
No/not reported	456	4 (2, 9)	
Yes	347	6 (2, 11)	
Presence of comorbidity			0.085
No/not reported	463	4 (2, 9)	
Yes	340	5 (2, 11)	

a Statistics presented: median (IQR).

b Statistical test: Kruskal–Wallis rank-sum test.

## Discussion

Our study reports a total of 46 870 confirmed COVID-19 and 803 deaths from eight countries in the WHO African region during the period 21 March to 31 October 2020. The key risk factors identified for mortality were male sex, older age, presence of one or more comorbidities and residence in capital cities. Of all reported confirmed cases, 2.9% were among healthcare workers, with a higher proportion of these cases being female (56.6%), with, however, an increased CFR in male healthcare workers. Being a healthcare worker was not attributed as a risk factor for mortality or time to death.

Monitoring the occurrence of deaths during a pandemic, and factors influencing this mortality, not only helps track the evolution of the pandemic but also helps decision makers target, prioritise and monitor the effectiveness of prevention and response strategies [18]. Although the African region accounts for <2.5% of COVID-19-associated deaths reported globally, it is crucial to understand the patterns of these deaths, the areas and populations affected, and identify the risk factors for death, in order to guide decision makers at national and regional levels.

Our observed overall incidence and CFR were on the lower end of the scale compared to several regions reporting hundreds of cases per 100 000 population and CFRs several times higher [11, 19]. The highest incidence observed in Sao Tome and Principe (42.02 per 100 000) and Eswatini (11.21 per 100 000) could be explained by the small size and high density population [20, 21]. The overall low case fatality observed in our study makes the region among the least affected. Studies in the early stages of the pandemic suggest that lockdown may have delayed epidemics by about 3 months [22]. It is important to note that the crude (total cases prior to inclusion criteria) estimate of incidence presented here for the same time period is double what we report, with the overall CFR also being slightly higher. This suggests that our observations may be underestimating the true state of the COVID-19 situation in the region. It should also be considered that CFRs can be influenced by the bias from possible discrepancies in per capita testing rates between the African countries included in this study.

Three key risk factors have stood out to date with relation to COVID-19-associated deaths, namely age, sex and the presence of comorbidities. Our observed incidence and CFR by age and sex aligned with other studies, with older age and being male being widely documented as key risk factors, with higher mortality among older males [23, 24]. Persons over the age of 65 years have been shown to have a 62 times higher mortality rate than those under 55 years [25]. In our data, we find that those over 60 have a CFR 10 times greater than those 60 and under and 15 times greater than those under 50, demonstrating that the same dynamics hold true despite the different population age structure of countries in the WHO African region. However, it is possible that new variants, arising in later stages of the pandemic, could result in the shifting of this age demographic, and should be investigated in further studies

Persons with non-communicable diseases, such as cardiovascular disease and diabetes, have also been identified as having greater risk of COVID-19-associated mortality, with the risk of death increasing with the number of comorbidities [26, 27]. In a study of causes of deaths in South Africa during the first 99 days of the pandemic, individuals with two or more comorbidities accounted for 58.6% of these deaths, with hypertension and diabetes the most commonly reported diseases [28].

Our study found that cardiovascular disease, diabetes and hypertension were the most commonly reported comorbidities among cases in the countries studied in the WHO African region. Having any comorbidity increased the hazard of death 12-fold, reaching a maximum of 66-fold for cases with four comorbidities (the maximum reported). This is again in keeping with the globally identified mortality risk COVID-19 poses to those with comorbidities [29]. With the absolute burden of non-communicable diseases in Africa being comparable to other regions [30], our findings suggest that we should not disregard the impact of COVID-19 on people with non-communicable diseases, and *vice versa*, in the WHO African region. It is key for public health authorities in the WHO African region to address the growing burden of non-communicable diseases as part of the COVID-19 response, as suggested previously by the WHO [31]. Affordable and proven cost-effective interventions should be made available to countries to prevent and manage non-communicable diseases in the context of COVID-19. Critically, interruptions in non-communicable disease services that have occurred as a result of diversion of resources to COVID-19 responses must be addressed. Some countries have already started, with alternative strategies such as triaging and telemedicine, while continuity of non-communicable diseases services has been ensured by others in their list of essential health services [31].

Several studies have reported higher COVID-19 incidence among healthcare workers, however few have reported on differences in mortality [32, 33]. Healthcare workers have a lower hazard in our study, which may be explained by the fact that there were more young female healthcare workers captured in our data. It should be noted however, that our measures involving healthcare workers may be subject to potential bias, possibly due to ease of testing access within this setting.

Spatial disparities have been reported as an independent risk factor for infection-related mortality when comparing metropolitan and non-metropolitan areas [20]. The reasoning behind this is linked to high-population density and opportunities for increased transmission through more socio-economic interactions. Some studies have found that COVID-19 deaths are concentrated in large cities and surrounding metropolitan areas. However, small cities or rural communities were also found to have equivalently high rates when opportunities for large gatherings, such as funerals, presented themselves and there was an infected individual(s) in attendance [21]. Our findings reflect the latter, and in addition our data suggest that living outside a capital city significantly decreases time to death.

The time to death for COVID-19 has been shown to vary widely across studies and has been linked to age and presence of comorbidities [34, 35]. To our knowledge, no other studies report on time from having a positive test to death. Several studies report on the (more clinically useful) time from hospital admission of COVID-19 patients to death [36, 37], which could have impacted the lack of association found with time to death and comorbidity presence. Though not directly comparable, our median times being lower than the studies mentioned may be suggestive of limited critical resources and access to early supportive care in some countries which may lead to accelerated deterioration [38].

### Study limitations

Several important limitations to this analysis should be noted, most importantly the generalisability of these results. The low number of countries included (8 of 47 countries in the WHO African region) means that our results are not representative of the whole African region. In addition, it is not possible to make valid comparisons between countries due to likely differences in testing and reporting of cases and associated deaths. Most countries, despite reporting up until the 31 October, did not report confirmed cases right up to that date. This means that for those countries we may be underestimating CFR for the period, and thereby also for the overall CFR estimate. We must also note the weaknesses of the civil registration system, with death reporting universally adopted in only eight countries of the African continent [39].

Cases dropped due to missing or incorrect date variables, as well as those missing information on age and sex, may have further contributed to CFR inaccuracy (under or overdepending on whether/how systematically missing). Beyond the issues in data collection, the choice to interpret ‘not reported’ as ‘no’ for exposure variables may have led to inaccurate stratified CFRs and may also have resulted in inaccurate aHR estimates again depending on the pattern of missingness.

Our regressions only include information on a few variables, meaning that we are likely missing a multitude of important confounders and effect modifiers; thus the results of multivariable regressions should be interpreted with caution.

We acknowledge the association with comorbidities in our study may be due to reporting bias, because those who are the most ill will be more likely to be in hospital and thereby more likely to be captured, tested and reported. However, when considering that we included specific comorbidities of interest, it is possible that the association with non-communicable disease comorbidities would be even higher had this information been systematically collected. It should also be considered that differences in disease burden between countries within the African region may influence our findings. It is also possible that our observations on those residing in capital cities are due to reporting bias, as there will be higher clinical, testing and public health capacity in capital cities. However, this could also mean our observation for time to death is valid due to the disparities in healthcare availability and capacity in rural and urban areas. In addition, selection bias may also influence measures surrounding healthcare workers, due to ease of testing access within this setting. This may result in an over-representation of healthcare workers in our sample.

Finally, our analysis has only captured people who have a confirmed positive test, and so are only informative at the population level hazard of death associated with a positive diagnosis of COVID-19. This is an important limitation for both clinical and public health decisions making. Future studies that either have more complete data (in terms of both countries included, cases captured and information collected), or are designed to investigate specific risk factors in clinical settings and within the specific African region countries, may provide more comprehensive information for the region.

## Conclusion

This study is, to our knowledge, the only study of its size that investigates the mortality burden and risk factors for COVID-19 in the WHO African region. The utility of analysing observational data for decision making, as opposed to relying solely on assumption-based mathematical models, cannot be understated. Our study found that the overall incidence and CFR were on the lower end of the scale compared to several regions reporting hundreds of cases per 100 000 population and CFRs several times higher, but this may be due to under-reporting. Four key risk factors were associated with mortality, namely male sex, older age, presence of one or more comorbidities and residence in capital cities.

Mortality from COVID-19 in Africa is likely to be comparable with that elsewhere, although under-reported, with many of the same risk factors for this present in these populations. The incidence of non-communicable diseases across the region is also comparable to other, perhaps better studied, regions. This makes it important to consider these diseases in future studies and in health system and future pandemic planning.

## Data Availability

The data that support the findings of this study are available on request from the corresponding author (BI). Some of the data are publicly available through situation reports produced by Ministries of Health and WHO/AFRO on their respective websites.

## Supplementary material

For supplementary material accompanying this paper visit https://doi.org/10.1017/S095026882100251X. click here to view supplementary material

## References

[1] World Health Organisation (WHO). Novel Coronavirus – China. WHO. World Health Organization. Available at http://www.who.int/csr/don/12-january-2020-novel-coronavirus-china/en/ (Accessed 26 January 2021).

[2] Petersen E (2020) Comparing SARS-CoV-2 with SARS-CoV and influenza pandemics. The Lancet Infectious Diseases 20, e238–e244. 32628905 10.1016/S1473-3099(20)30484-9 PMC7333991

[3] World Health Organization (2003) Consensus Document on the Epidemiology of Severe Acute Respiratory Syndrome (SARS). Geneva: World Health Organization.

[4] World Health Organisation EMRO. *MERS situation update, January 2020*. Available at http://www.emro.who.int/pandemic-epidemic-diseases/mers-cov/mers-situation-update-january-2020.html (Accessed 22 January 2021).

[5] World Health Organisation (WHO). *WHO Coronavirus (COVID-19) Dashboard*. Available at https://covid19.who.int (Accessed 30 September 2021).

[6] Breban R, Riou J and Fontanet A (2013) Interhuman transmissibility of Middle East respiratory syndrome coronavirus: estimation of pandemic risk. Lancet 382, 694–699. 23831141 10.1016/S0140-6736(13)61492-0 PMC7159280

[7] WHO Regional Office for Africa (2020) *A second COVID-19 case is confirmed in Africa*. *WHO|Regional Office for Africa*. Available at https://www.afro.who.int/news/second-covid-19-case-confirmed-africa (Accessed 21 January 2021).

[8] WHO Regional Office for Africa. *Situation reports on COVID-19 outbreak – Sitrep 11, 13 May 2020*. *WHO|Regional Office for Africa*. Available at https://www.afro.who.int/publications/situation-reports-covid-19-outbreak-sitrep-11-13-may-2020 (Accessed 26 January 2021).

[9] Ghosh D, Bernstein JA and Mersha TB (2020) COVID-19 pandemic: the African paradox. Journal of Global Health 10, 020348. 10.7189/jogh.10.020348 PMC7506193 33110546

[10] Mougeni F, Mangaboula A and Lell B (2020) The potential effect of the African population age structure on COVID-19 mortality. medRxiv. doi: 2020.05.19.20106914.

[11] Sajadi MM (2020) Temperature, humidity, and latitude analysis to estimate potential spread and seasonality of coronavirus disease 2019 (COVID-19). JAMA Network Open 3. Published online: 11 June 2020. doi: 10.1001/jamanetworkopen.2020.11834. PMC7290414 32525550

[12] Achoki T (2020) COVID-19 pandemic in the African continent: forecasts of cumulative cases, new infections, and mortality. medRxiv. doi: 2020.04.09.20059154.

[13] World Health Organisation (WHO) (2019) *Public health surveillance for COVID-19: interim guidance*. Available at https://www.who.int/publications-detail-redirect/who-2019-nCoV-surveillanceguidance-2020.8 (Accessed 28 January 2021).

[14] World Health Organisation (WHO) (2016) International Health Regulations (2005), 3rd Edn. Geneva: World Health Organisation.

[15] United Nations Department of Economic and Social Affairs. *World population prospects – population division – United Nations*. Available at https://population.un.org/wpp/ (Accessed 21 January 2021).

[16] World Health Organisation (WHO) *Estimating mortality from COVID-19*. Available at https://www.who.int/news-room/commentaries/detail/estimating-mortality-from-covid-19 (Accessed 21 January 2021).

[17] Dunkler D (2018) Weighted cox regression using the R package coxphw. Journal of Statistical Software 84, 1–26.30450020

[18] Setel P (2020) Mortality surveillance during the COVID-19 pandemic. Bulletin of the World Health Organization 98, 374–374. 32514207 10.2471/BLT.20.263194 PMC7265935

[19] *WHO Coronavirus Disease (COVID-19) Dashboard*. Available at https://covid19.who.int (Accessed 26 January 2021).

[20] Bhadra A, Mukherjee A and Sarkar K (2020) Impact of population density on Covid-19 infected and mortality rate in India. Modeling Earth Systems and Environment. Published online: 14 October 2020. doi: 10.1007/s40808-020-00984-7. PMC7553801 33072850

[21] Stier A, Berman MG and Bettencourt L (2020) COVID-19 attack rate increases with city size. Published online: 30 March 2020.

[22] van Zandvoort K (2020) Response strategies for COVID-19 epidemics in African settings: a mathematical modelling study. BMC Medicine 18, 324. 33050951 10.1186/s12916-020-01789-2 PMC7553800

[23] Peckham H (2020) Male sex identified by global COVID-19 meta-analysis as a risk factor for death and ITU admission. Nature Communications 11, 6317. 10.1038/s41467-020-19741-6 PMC7726563 33298944

[24] O'Driscoll M (2020) Age-specific mortality and immunity patterns of SARS-CoV-2. Nature 590, 1–6. 10.1038/s41586-020-2918-0 33137809

[25] Yanez ND (2020) COVID-19 mortality risk for older men and women. BMC Public Health 20, 1742. 33213391 10.1186/s12889-020-09826-8 PMC7675386

[26] Ssentongo P (2020) Association of cardiovascular disease and 10 other pre-existing comorbidities with COVID-19 mortality: a systematic review and meta-analysis. PLoS ONE 15, e0238215. 32845926 10.1371/journal.pone.0238215 PMC7449476

[27] Harrison SL (2020) Comorbidities associated with mortality in 31,461 adults with COVID-19 in the United States: a federated electronic medical record analysis. PLoS Medicine 17, e1003321. 32911500 10.1371/journal.pmed.1003321 PMC7482833

[28] Wyk VP (2020) COVID deaths in South Africa: 99 days since South Africa's first death. South African Medical Journal 110, 1093–1099. 33403985

[29] Horton R (2020) Offline: COVID-19 is not a pandemic. The Lancet 396, 874. 10.1016/S0140-6736(20)32000-6 PMC7515561 32979964

[30] GBD 2019 Diseases and Injuries Collaborators (2020) Global burden of 369 diseases and injuries in 204 countries and territories, 1990–2019: a systematic analysis for the Global Burden of Disease Study 2019. Lancet 396, 1204–1222. 33069326 10.1016/S0140-6736(20)30925-9 PMC7567026

[31] World Health Organisation (WHO) (2020) The Impact of the COVID-*19* Pandemic on Noncommunicable Disease Resources and Services: Results of a Rapid Assessment. Geneva: World Health Organisation.

[32] Office for National Statistics (2020) *Which occupations have the highest potential exposure to the coronavirus (COVID-19)?* Available at https://www.ons.gov.uk/employmentandlabourmarket/peopleinwork/employmentandemployeetypes/articles/whichoccupationshavethehighestpotentialexposuretothecoronaviruscovid19/2020–05–11 (Accessed 22 January 2021).

[33] Bandyopadhyay S (2020) Infection and mortality of healthcare workers worldwide from COVID-19: a systematic review. BMJ Global Health 5, e003097. 10.1136/bmjgh-2020-003097 PMC7722361 33277297

[34] Verity R (2020) Estimates of the severity of coronavirus disease 2019: a model-based analysis. The Lancet Infectious Diseases 20, 669–677. 32240634 10.1016/S1473-3099(20)30243-7 PMC7158570

[35] Alaa A (2020) Retrospective cohort study of admission timing and mortality following COVID-19 infection in England. BMJ Open 10, e042712. 10.1136/bmjopen-2020-042712 PMC7684820 33234660

[36] Faes C (2020) Time between symptom onset, hospitalisation and recovery or death: statistical analysis of Belgian COVID-19 patients. International Journal of Environmental Research and Public Health 17. Published online: October 2020. doi: 10.3390/ijerph17207560. PMC7589278 33080869

[37] Azoulay E (2020) Increased mortality in patients with severe SARS-CoV-2 infection admitted within seven days of disease onset. Intensive Care Medicine 46, 1714–1722. 32780165 10.1007/s00134-020-06202-3 PMC7417780

[38] Asiimwe E and Saraswati K (2020) Covid-19 and sub-Saharan Africa's critical care infrastructure. BMJ. Available at https://blogs.bmj.com/bmj/2020/04/23/covid-19-and-sub-saharan-africas-critical-care-infrastructure/ (Accessed 28 January 2021).

[39] United Nations (2017) Economic Commission for Africa. Report on the status of civil registration and vital statistics in Africa: outcome of the Africa programme on accelerated improvement of civil registration and vital statistics systems monitoring framework. *Addis Ababa. © UN. ECA*. Published online: November 2017.

