# Supplementary material for: Time to death and risk factors associated with mortality among COVID-19 cases in countries within the WHO African region in the early stages of the COVID-19 pandemic
Source: Epidemiol Infect. 2022 Feb 18;150:e73. doi: 10.1017/S095026882100251X (PMC9002149; doi:10.1017/S095026882100251X)
Supplement: Supplementary file 1 [file hygsup.zip › S095026882100251Xsup001.docx]

**SUPPLEMENTARY MATERIAL**

Supplementary Table 1: characteristics and missingness for variables of interest among confirmed cases reported in 8 Member States of the WHO African region between 21 March and 31 October 2020 (N =46870)

| **Characteristic** | **N = 46870*** | **Dead, N = 803 (1.7%)^†^** |  |
| --- | --- | --- | --- |
| Sex |  |  |  |
| *Female* | 17012 (36%) | 221 (1.3%) |  |
| *Male* | 29858 (64%) | 582 (1.9%) |  |
| Age | 35 (27, 46) | 58 (47, 69) |  |
| Health Care Worker |  |  |  |
| *No* | 13808 (29%) | 213 (1.5%) |  |
| *Not reported* | 31681 (68%) | 571 (1.8%) |  |
| *Yes* | 1381 (2.9%) | 19 (1.4%) |  |
| Residence in capital city |  |  |  |
| *No* | 8919 (19%) | 382 (4.3%) |  |
| *Not reported* | 24686 (53%) | 74 (0.3%) |  |
| *Yes* | 13265 (28%) | 347 (2.6%) |  |
| Presence of comorbidity |  |  |  |
| *No* | 14851 (32%) | 102 (0.7%) |  |
| *Not reported* | 29158 (62%) | 361 (1.2%) |  |
| *Yes* | 2861 (6.1%) | 340 (12%) |  |
| *Statistics presented: n (%); Median (IQR) | | | |
| †Statistic presented: n (Case Fatality Rate (%)); Median (IQR) | | | |
