# Supplementary material for: Time to death and risk factors associated with mortality among COVID-19 cases in countries within the WHO African region in the early stages of the COVID-19 pandemic
Source: Epidemiol Infect. 2022 Feb 18;150:e73. doi: 10.1017/S095026882100251X (PMC9002149; doi:10.1017/S095026882100251X)
Supplement: Supplementary file 1 [file hygsup.zip › S095026882100251Xsup002.docx]

Supplementary Table 2: Summary measures for COVID-19 cases reported between 21 March and 31 October 2020 by 11 WHO African region Member State, reporting up until and including 31 October, before further inclusion criteria for analysis applied (See Figure 1) (N=116304)

| **Country** | **Cases^*^ (N)** | **Recovered^†^ (n)** | **Dead (n)** | **Alive (n)^‡^** | **CFR (%)** | **Healthcare worker cases (N)** | **Healthcare worker deaths (n)** | **Healthcare worker cases (%)** | **CFR among healthcare workers (%)** | **Incidence per 100000** |
| --- | --- | --- | --- | --- | --- | --- | --- | --- | --- | --- |
| *Burkina Faso* | 1461 | 0 | 62 | 807 | 4.24 | 143 | 0 | 9.79 | 0 | 0.70 |
| *Democratic Republic of Congo* | 11351 | 551 | 481 | 10870 | 4.24 | 221 | 14 | 1.95 | 6.33 | 1.27 |
| *Guinea* | 12333 | 0 | 73 | 12260 | 0.59 | 775 | 7 | 6.28 | 0.9 | 9.39 |
| *Kenya* | 55867 | 6972 | 1,013 | 6974 | 1.81 | 1679 | 19 | 3.01 | 1.13 | 10.39 |
| *Mauritius* | 432 | 22 | 8 | 424 | 1.85 | 26 | 1 | 6.02 | 3.85 | 3.40 |
| *Namibia* | 12761 | 607 | 70 | 12546 | 0.55 | 535 | 2 | 4.19 | 0.37 | 50.22 |
| *Niger* | 1217 | 1035 | 90 | 1127 | 7.4 | 178 | 0 | 14.63 | 0 | 0.50 |
| *Sao Tome and Principe* | 948 | 0 | 16 | 932 | 1.69 | 0 | 0 | 0 |  | 43.26 |
| *Eswatini* | 5916 | 2594 | 114 | 2594 | 1.93 | 321 | 3 | 5.43 | 0.93 | 50.99 |
| *Chad* | 1275 | 18 | 94 | 1181 | 7.37 | 1 | 0 | 0.08 | 0 | 0.78 |
| *Uganda* | 12743 | 0 | 145 | 12597 | 1.14 | 77 | 1 | 0.6 | 1.3 | 2.79 |
| **Total** | **116304** | **11799** | **2166** | **62312** | **1.86** | **3956** | **47** | **3.40** | **1.19** | **43.25** |
| *Confirmed cases reported 21^st^ March-31^st^ October 2020  †Recovery status missing for Sao Tome and Principe and Uganda  ‡ Recovered cases are included in Alive count. Alive and dead sum to total cases. | | | | | | | | | | |
