# Supplementary material for: Time to death and risk factors associated with mortality among COVID-19 cases in countries within the WHO African region in the early stages of the COVID-19 pandemic
Source: Epidemiol Infect. 2022 Feb 18;150:e73. doi: 10.1017/S095026882100251X (PMC9002149; doi:10.1017/S095026882100251X)
Supplement: Supplementary file 1 [file hygsup.zip › S095026882100251Xsup003.docx]

Supplementary Table 3: Age and sex distribution of confirmed cases and deaths among healthcare workers reported by 8 Member States of the WHO African region between 21 March and 31 October 2020 (N=1381)

| **Age Group** | **Cases^*^(N= 1381)^†^** | | **Dead (n=19)^†^** | | **CFR (%)**  **(overall: 1.38%)** | |
| --- | --- | --- | --- | --- | --- | --- |
|  | ***F*** | ***M*** | ***F*** | ***M*** | ***F*** | ***M*** |
| < 15 years | 1 (0.12%) | 0 (0%) | 0 (0%) | 0 (0%) | 0.00 | 0.00 |
| 15-24 years | 47 (6%) | 27 (4.5%) | 0 (0%) | 0 (0%) | 0.00 | 0.00 |
| 25-34 years | 321 (41%) | 192 (32%) | 1 (17%) | 0 (0%) | 0.00 | 0.00 |
| 35-44 years | 196 (25%) | 182 (30%) | 0 (0%) | 3 (23%) | 0.00 | 0.02 |
| 45-54 years | 142 (18%) | 115 (19%) | 1 (17%) | 2 (15%) | 0.01 | 0.02 |
| 55-64 years | 70 (8.9%) | 66 (11%) | 3 (50%) | 2 (15%) | 0.04 | 0.03 |
| 65-74 years | 5 (0.63%) | 11 (1.8%) | 1 (17%) | 4 (30%) | 0.20 | 0.36 |
| 75 years + | 1 (0.12) | 5 (0.83%) | 0 (0%) | 2 (15%) | 0.00 | 0.40 |
| ***Total*** | ***783*** | ***598*** | ***6*** | ***13*** | ***0.77*** | ***2.17*** |
| *****Confirmed cases reported 21^st^ March-31^st^ October 2020  †Statistics presented: n (% within sex total) | | | | | | |
