# Supplementary material for: Time to death and risk factors associated with mortality among COVID-19 cases in countries within the WHO African region in the early stages of the COVID-19 pandemic
Source: Epidemiol Infect. 2022 Feb 18;150:e73. doi: 10.1017/S095026882100251X (PMC9002149; doi:10.1017/S095026882100251X)
Supplement: Supplementary file 1 [file hygsup.zip › S095026882100251Xsup004.docx]

Supplementary Table 4: Condition specific case fatality ratio among confirmed cases with conditions of interest in 8 Member States of the WHO African region between 21 March and 31 October 2020 (N =46870) (Note: cases with multiple conditions are counted for each condition).

| **Condition** | **Total cases (N)** | **Dead (N)** | **Alive (N)** | **CFR (%)** |
| --- | --- | --- | --- | --- |
| Renal Disease | 60 | 27 | 33 | 45.00 |
| Hypertension | 535 | 184 | 351 | 34.39 |
| Cancer | 51 | 14 | 37 | 27.45 |
| Tuberculosis | 45 | 12 | 33 | 26.67 |
| Diabetes | 585 | 118 | 467 | 20.17 |
| Asthma | 142 | 14 | 128 | 9.86 |
| Not Specified | 102 | 9 | 93 | 8.82 |
| Obesity | 138 | 10 | 128 | 7.25 |
| Chronic Pulmonary Disease | 238 | 17 | 221 | 7.14 |
| Other | 712 | 48 | 664 | 6.74 |
| Cardiovascular Disease | 951 | 39 | 912 | 4.1 |
| Pregnancy^*^ | 153 | 2 | 151 | 1.31 |
| Drepanocytosis | 4 | 0 | 4 | 0 |
| *Demonstrated as a risk factor, not included as a comorbidity | | | | |
