# Supplementary material for: Time to death and risk factors associated with mortality among COVID-19 cases in countries within the WHO African region in the early stages of the COVID-19 pandemic
Source: Epidemiol Infect. 2022 Feb 18;150:e73. doi: 10.1017/S095026882100251X (PMC9002149; doi:10.1017/S095026882100251X)
Supplement: Supplementary file 1 [file hygsup.zip › S095026882100251Xsup005.docx]

Supplementary Table 5: Unweighted cox regression for mortality by various characteristics among confirmed cases reported in 8 Member States in the WHO African region between 21 March and 31 October 2020 (N = 46870)

|  | **Univariate** | | | | **Multivariable** | | |
| --- | --- | --- | --- | --- | --- | --- | --- |
| Characteristic | N | HR^*^ | 95% CI^*^ | p-value | aHR^*^ | 95% CI^*^ | p-value |
| Sex (Male) | 46870 | 1.50 | 1.29, 1.75 | <0.001 | 1.55 | 1.33, 1.81 | <0.001 |
| Age | 46870 | 1.08 | 1.07, 1.08 | <0.001 | 1.08 | 1.07, 1.08 | <0.001 |
| Health Care Worker | 46870 | 0.77 | 0.49, 1.22 | 0.3 | 0.60 | 0.38, 0.95 | 0.031 |
| Residence in capital city | 46870 | 1.87 | 1.63, 2.15 | <0.001 | 1.41 | 1.22, 1.62 | <0.001 |
| Presence of comorbidity | 46870 | 11.7 | 10.2, 13.5 | <0.001 | 32.8 | 19.3, 55.7 | <0.001 |
| Pregnancy | 17012 | 0.99 | 0.25, 3.97 | >0.9 |  |  |  |
| Presence of comorbidity * Age |  |  |  |  | 0.97 | 0.96, 0.98 | <0.001 |
| ^*^HR = Hazard Ratio, CI = Confidence Interval aHR= adjusted Hazard Ratio  ^†^Females only | | | | | | | |
